# Cotinine halts the advance of Alzheimer's disease-like pathology and associated depressive-like behavior in Tg6799 mice

**DOI:** 10.3389/fnagi.2014.00162

**Published:** 2014-07-23

**Authors:** Sagar Patel, J. Alex Grizzell, Rosalee Holmes, Ross Zeitlin, Rosalynn Solomon, Thomas L. Sutton, Adeeb Rohani, Laura C. Charry, Alexandre Iarkov, Takashi Mori, Valentina Echeverria Moran

**Affiliations:** ^1^Research and Development Service, Department of Veterans Affairs, Bay Pines VA Healthcare SystemBay Pines, FL, USA; ^2^Department of Psychiatry and Behavioral Neurosciences, Morsani College of Medicine, University of South FloridaTampa, FL, USA; ^3^Center of Research in Biomedical Sciences, Universidad Autónoma de ChileSantiago, Chile; ^4^Departments of Biomedical Sciences and Pathology, Saitama Medical Center and Saitama Medical UniversityKawagoe, Saitama, Japan; ^5^Research Service, Department of Veterans Affairs, Tampa VA Healthcare SystemTampa, FL, USA; ^6^Department of Molecular Medicine, Morsani College of Medicine, University of South FloridaTampa, FL, USA

**Keywords:** Alzheimer's disease, amyloid-β, cotinine, depression, memory loss, postsynaptic density protein 95, protein kinase B, Akt

## Abstract

Alzheimer's disease (AD) is associated with cognitive and non-cognitive symptoms for which there are currently no effective therapies. We have previously reported that cotinine, a natural product obtained from tobacco leaves, prevented memory loss and diminished amyloid-β (Aβ) plaque pathology in transgenic 6799 mice (Tg6799 mice) when treated prior to the development of the pathology. We have also shown that cotinine reduces depressive-like behavior in normal and chronically stressed C57BL/6 mice. Here, we extend our previous studies by investigating the effects of cotinine on the progression of AD-like pathology, depressive-like behavior, and the mechanisms underlying its beneficial effects in Tg6799 mice when left untreated until after a more advanced stage of the disease's development. The results show that vehicle-treated Tg6799 mice displayed an accentuated loss of working memory and an abundant Aβ plaque pathology that were accompanied by higher levels of depressive-like behavior as compared to control littermates. By contrast, prolonged daily cotinine treatment to Tg6799 mice, withheld until after a mid-level progression of AD-like pathology, reduced Aβ levels/plaques and depressive-like behavior. Moreover, this treatment paradigm dramatically improved working memory as compared to control littermates. The beneficial effects of cotinine were accompanied by an increase in the expression of the active form of protein kinase B and the postsynaptic density protein 95 in the hippocampi and frontal cortices of Tg6799 mice. This suggests that cotinine halts the progression of AD-like pathology while reducing depressive-like behavior by stimulating signaling pathways supporting synaptic plasticity in Tg6799 mice. The potential use of cotinine to treat cognitive and non-cognitive symptoms of AD is discussed.

## Introduction

Alzheimer's disease (AD) is a devastating neurodegenerative disease and the major cause of dementia worldwide (Stone et al., 2011). It is estimated that well over 25 million people suffer from dementia today with one new case diagnosed every 7 s (Ferri et al., 2005). The number of individuals affected in developed countries are expected to double in the United States and triple in both India and China as well as their south Asian and western pacific neighbors over the next three decades (Ferri et al., 2005). Drugs currently used to treat AD, such as acetylcholinesterase (AChE) inhibitors and the N-methyl-D-aspartate (NMDA) antagonists, have only modest effects in delaying the progression of the disease (Starkstein and Mizrahi, 2006; Hansen et al., 2007; Raina et al., 2008). Moreover, the tolerability of these drugs is compromised by their side effects, for instance dizziness, anorexia, vomiting, and diarrhea (Alva and Cummings, 2008).

In patients suffering from AD, there is a high incidence of depression (~50%) that precedes (Meynen et al., 2007; Modrego, 2010; Vilalta-Franch et al., 2013) and may accelerate the clinical evolution of the disease (Starkstein and Mizrahi, 2006; Hou et al., 2010; Aznar and Knudsen, 2011; Raudino, 2013; Theleritis et al., 2013). Depression in AD is linked with earlier admission to nursing homes and increased mortality (Even and Weintraub, 2010). Unfortunately, commonly used antidepressants such as fluoxetine and sertraline (Weintraub et al., 2010) have only marginal short-term effects on depressive symptoms related to AD (Modrego, 2010). In addition, the alternative use of antipsychotics as an antidepressant in the elderly population has been questioned for safety reasons (Assal and van der Meulen, 2009; Gardette et al., 2012). The failure of these commonly used antidepressants can be explained by the fact that depression in AD patients has characteristics that differ from its expression in other conditions (Hollingworth et al., 2006). It is therefore imperative to investigate the effectiveness of novel drugs with better safety profiles to treat cognitive and non-cognitive symptoms in AD (Alva and Cummings, 2008). In doing so, such compounds can greatly decrease the extra burden in care-giving (Deimling and Bass, 1986), rates of institutionalization (Steele et al., 1990), and overall associated financial costs (Cohen-Mansfield, 1995). To the best of our knowledge, drugs reducing both cognitive and non-cognitive symptoms in AD have not been described.

We have previously shown that cotinine, a natural product obtained from tobacco leaves and the predominant metabolite of nicotine, improved memory and reduced amyloid-β (Aβ) plaque pathology in a transgenic 6799 mouse (Tg6799 mouse) model of AD when treatment began prior to the disease's development (Echeverria et al., 2011). Tg6799 mice express the human amyloid precursor protein (APP) and presenilin 1 (PS1) genes containing three familial AD (FAD) mutations in APP and two in PS1 (Oakley et al., 2006; Ohno et al., 2006). Cotinine also reduces depressive-like behavior in normal and chronically stressed C57BL/6 mice (Grizzell et al., 2014). Furthermore, cotinine stimulates protein kinase B (Akt) and inhibits the glycogen synthase kinase 3β (GSK3β) in the brains of stressed C57BL/6 (Grizzell et al., 2014) and Tg6799 mice (Echeverria et al., 2011). Here, we investigated the effect of cotinine on working memory and Aβ plaque deposition in the brains of Tg6799 mice when treatment was withheld until more advanced stages of the AD-like pathology. Additionally, we sought to determine if cotinine's aforementioned antidepressant effects in C57BL/6 mice extend to Tg6799 mice following only 10 days of treatment. To better understand the molecular mechanisms involved in cotinine's actions, we also investigated whether cotinine stimulates the Akt/postsynaptic density protein 95 (PSD95) pathway, which plays a central role in mediating synaptic plasticity improvement induced by antidepressants and cognitive enhancers. The new evidence described in this report suggests that cotinine may represent a therapeutic agent to treat cognitive and non-cognitive symptoms in AD.

## Materials and methods

### Animals

All experiments were performed using Tg6799 mice (5 × FAD) which express the human APP and PS1 genes, containing five FAD mutations (Oakley et al., 2006), three in APP (Swedish mutation: K670N, M671L; Florida mutation: I716V; London mutation: V717I) and two in PS1 (M146L, L286V) (Ohno et al., 2006). The Tg6799 line mice were maintained as hemizygotes on a C57BL/6J hybrid background (The Jackson Laboratories, Bar Harbor, ME). Male mice were used as heterozygotes with respect to the transgene and non-transgenic (NT) wild-type littermates served as controls. Mice were maintained on a 12:12 light-dark cycle with *ad libitum* access to food and water. Protocols were performed with the approval of the Institutional Animal Care and Use Committees of the University of South Florida and the Bay Pines Veterans Affairs Healthcare System.

### Cotinine treatment

Cotinine [(5*S*)-1-methyl-5-(3-pyridyl)-pyrrolidin-2-one] was obtained from Sigma-Aldrich (Saint Louis, MO). Mice began daily oral treatment with cotinine (5 mg/kg; Cot 5), dissolved in phosphate buffer saline (PBS, pH 7.4) or vehicle (PBS alone), *via* gavage between 4.5–5 months of age and were treated continuously until euthanasia. The dose used in this study was chosen based in our previous AD studies showing that a dose of cotinine 2.5 mg/kg prevented cognitive impairment and reduced Aβ plaque formation in Tg6799 mice (Zeitlin et al., 2012). From parallel studies testing the effect of cotinine on depressive-like behavior, we have observed that cotinine (5 mg/kg) consistently decreased depressive-like behavior in wild-type mice as well as in several models of psychological stress (Grizzell et al., 2014). Thus, to target both cognitive and non-cognitive symptoms, we have tested this dose Cot 5 mg/kg in mice.

### Experimental conditions

#### Protocol 1

**Figure 1:**
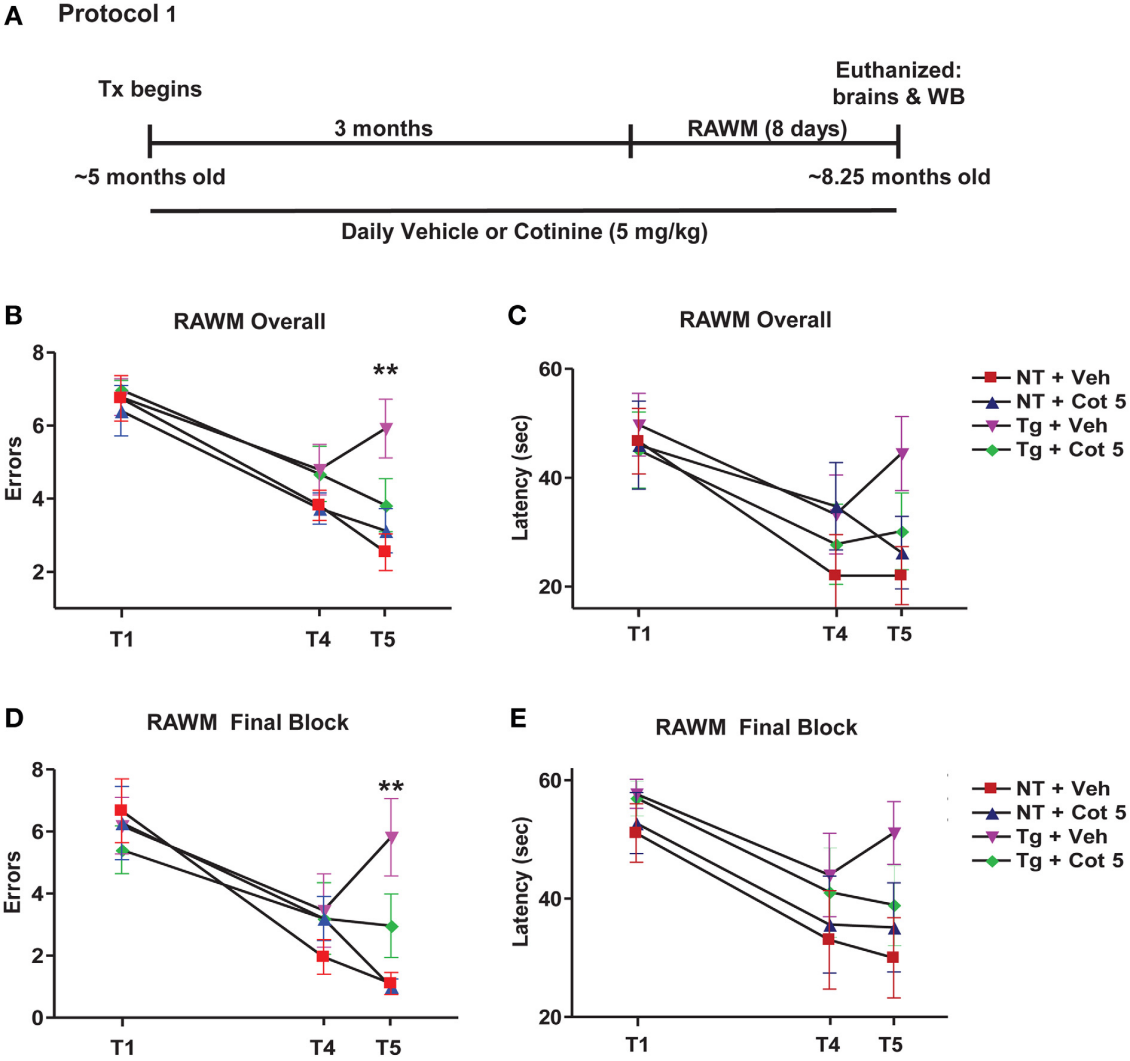
**Cotinine prevented cognitive impairment in Tg6799 mice.** 4.5–5 month-old mice (*n* = 8 per group) were treated daily for 3 months and tested in the radial arm water maze (RAWM; **A**). Chronic cotinine treatment (5 mg/kg) diminished cognitive impairment in the transgenic (Tg) mice as assessed using the RAWM testing for working memory. This tests revealed that cotinine-treated Tg mice (Tg + Cot 5) made fewer errors than vehicle-treated Tg mice (Tg + Veh) when tested overall (^**^p < 0.01; **B**) and during the final block of testing (^**^p < 0.01; **D**). Latencies between groups overall **(C)** and during the final block **(E)** are also shown. Tx, treatment; WB, Western blot.

Tg6799 mice and NT littermates (~5 months of age) were treated with cotinine or vehicle (*n* = 8 per group) for 3 months and tested for spatial/working memory deficits in the radial arm water maze (RAWM) (Arendash et al., 2009; Grizzell et al., 2014) for 8 consecutive days. Following RAWM testing, mice were euthanized and brains were collected for Western blot analysis (Figure 1A).

#### Protocol 2

**Figure 2:**
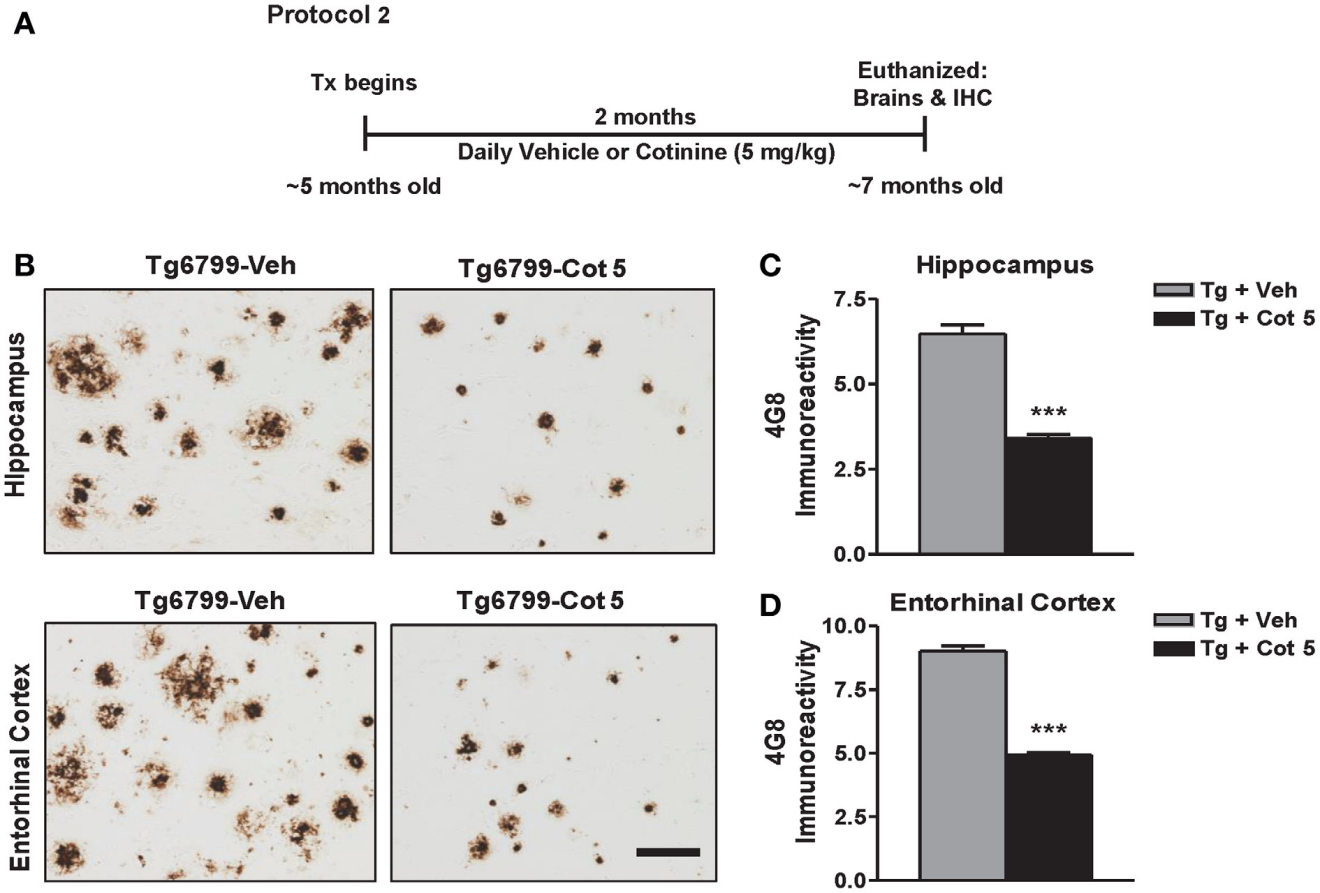
**Cotinine reduces Aβ plaques in the hippocampus and entorhinal cortex of Tg6799 mice.** Transgenic (Tg) mice at 4.5–5 months of age (*n* = 8 per group) were treated daily for 2 months with vehicle (Veh) or Cotinine (5 mg/kg; Cot 5), euthanized and Aβ immunoreactivity (IR) in the hippocampus and entorhinal cortex was measured by immunohistochemistry (IHC; **A**). Tg mice treated with cotinine (Tg + Cot 5) showed less Aβ plaques in the hippocampus and entorhinal cortex compared to vehicle-treated Tg mice (Tg + Veh; **B**). The photomicrographs to the left are representative views of Aβ plaques in the hippocampus (*Upper*) and entorhinal cortex (*Lower*) of brain sections of Tg mice stained with the Aβ-specific antibody 4G8 (Scale bar = 50 μm). The graphs to the right represent the percentage of Aβ plaque burden in these brain regions. In the hippocampus, a 47% reduction (*t* = 10.03; ^***^p < 0.001; **C**) and in the entorhinal cortex, a 45% reduction (*t* = 16.47; ^***^p < 0.001; **D**) was found. Tx, treatment.

Tg6799 mice and NT littermates (~5 months of age) were treated for 2 months with cotinine or vehicle (*n* = 8 per group), euthanized, and brains were collected for immunohistochemical (IHC) analysis (Figure 2A).

#### Protocol 3

**Figure 3:**
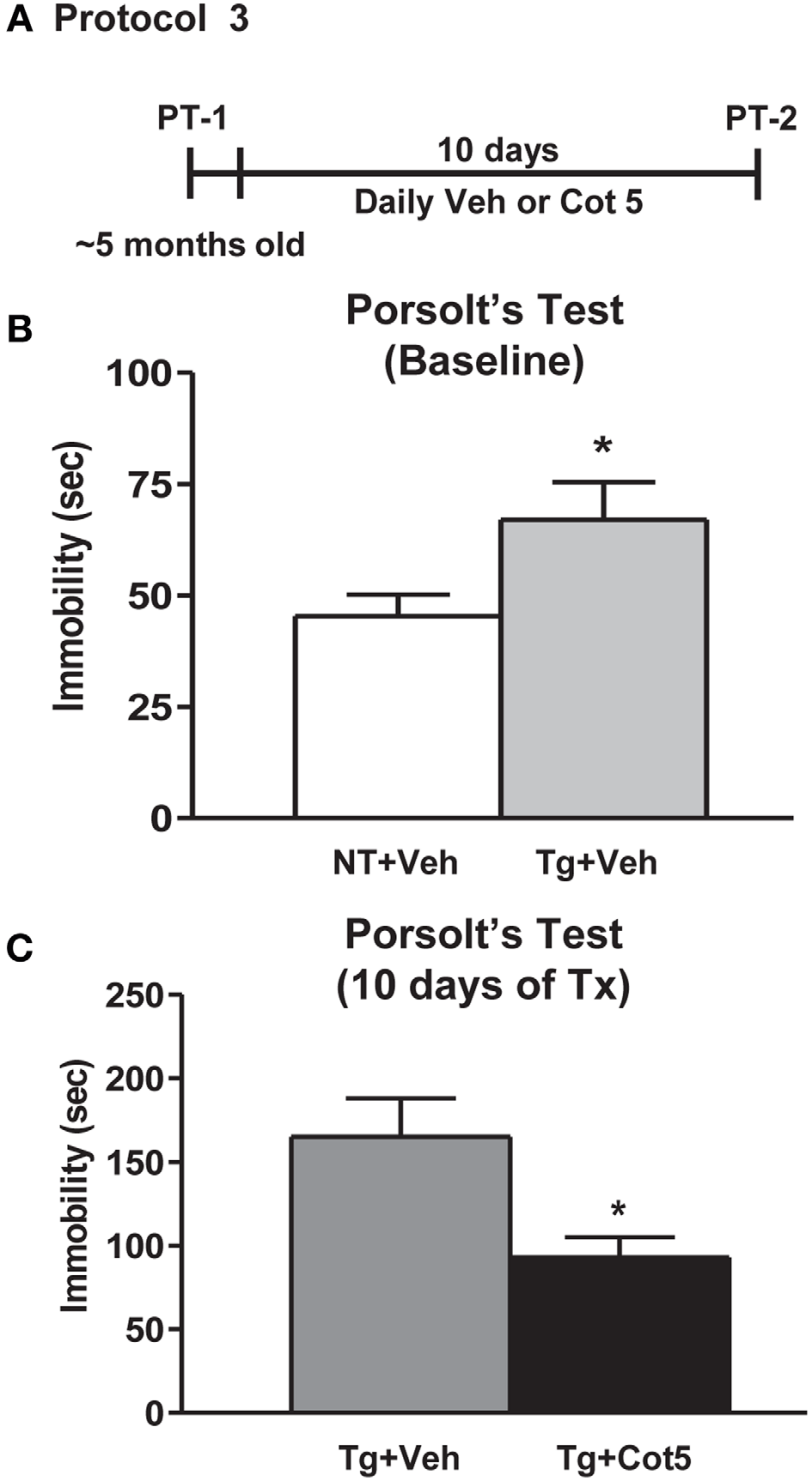
**Cotinine diminished depressive-like behavior in Tg6799 mice.** At 4.5–5 months of age, naïve transgenic (Tg) mice and non-transgenic (NT) littermates (*n* = 16 per group) were tested for basal levels of depressive-like behavior in the Porsolt's forced swim test (PT-1; **A**). Tg mice displayed significantly greater immobility times than NT counterparts (*t* = 2.158, *df* = 30, ^*^p = 0.0391; **B,C**). Following 10 days of treatment with cotinine (5 mg/kg) or vehicle, Tg6799 mice were retested in the PT (PT-2). Cotinine-treated Tg mice (Tg + Cot 5) showed significantly less immobility than vehicle-treated Tg (Tg + Veh) cohorts (*t* = 2.775, *df* = 14, ^*^p = 0.0149). sec, seconds; Tx, treatment.

Tg6799 mice and NT littermates (~5 months of age; *n* = 16 per group) were tested for basal depressive-like behavior using Porsolt's forced swim test (PT; basal = PT-1). Following 10 days of cotinine or vehicle treatment in Tg6799 mice (*n* = 8 per group), the effects of cotinine on depressive-like behavior in the PT was tested again (PT-2; Figure 3A).

### Behavioral testing

#### Radial arm water maze (spatial working memory test)

The RAWM test consists of six radially distributed swim arms emanating from a central circular insert placed into a 100 cm circular pool and was conducted as previously described (Arendash et al., 2009; Grizzell et al., 2014). For mice in protocol 1, the latency and number of errors prior to locating the swim arm containing a submerged escape platform was determined for 5 trials per day over 8 days of testing. The latency and number of errors during Trial (T) 4 and T5, which are separated by a 30-min period, are both considered indices of working memory encoding and recall. The statistical analysis was carried out by analyzing performance over all 8 days and during the last 3 days (final block) of the RAWM test.

#### Porsolt's forced swim test (depressive-like behavior)

The PT is broadly used to assess depressive-like behavior (Naitoh et al., 1992). Mice from protocol 3 were placed in a transparent cylinder filled with water at 25°C for 5 min. Two investigators blind to all treatment levels independently scored immobility, which is considered a measure of depressive-like behavior in rodents (Naitoh et al., 1992; Karl et al., 2003). Immobility is herein defined as the summation of time when the mouse is considered motionless in the water, and includes only the movement necessary to keep its head above the water. Following testing, mice were hand-dried with a towel and returned to their home cages.

### Brain tissue preparation

For the protein and plaques analyses, mice from protocols 1 and 2 were euthanized and perfused with cold PBS (pH 7.4). The left frontal half of each brain was placed in 4% paraformaldehyde in PBS (pH 7.4) overnight, wherein tissues remained until the paraffin embedding process for Aβ IHC analyses. The rest of the brains were dissected out into regions of interest, quickly frozen, and stored at −80°C for neurochemical analyses.

### Aβ plaque analysis

Aβ plaques were analyzed in mice from protocol 2 as previously described (Arendash et al., 2009). The posterior cortex from the left side of the brain was dissected. Five coronal sections of paraffin blocks were sectioned at the level of hippocampus and entorhinal cortex (bregma −2.92 to −3.64 mm), according to Franklin and Paxinos (Franklin and Paxinos, 2001), and five 5-μ m sections (100 μm apart) were made from each mouse brain using a sliding microtome. IHC analyses were performed using a Vectastain ABC *Elite* kit (Vector Laboratories, Burlingame, CA) coupled with the diaminobenzidine reaction, except that the biotinylated secondary antibody step was omitted for Aβ IHC staining. A biotinylated human Aβ monoclonal antibody (clone 4G8, Covance Research Products, Emeryville, CA) was incubated for 1 h at room temperature. PBS (pH 7.4) or normal rabbit serum was used instead of primary antibody or ABC reagent as a negative control. Brain sections were treated with 70% formic acid prior to the pre-blocking step. Quantitative image analysis was conducted as previously described (Tan et al., 2002; Mori et al., 2006) with modifications. Images were acquired using an Olympus BX60 microscope with an attached digital camera system (DP-70, Olympus, Tokyo, Japan), and digital images were routed into a Windows PC for quantitative analysis using a SimplePCI software (Hamamatsu Photonics, Hamamatsu, Shizuoka, Japan). Images of three sections through both anatomic regions of interest (entorhinal cortex and CA1 region of the hippocampus) were captured from each animal, and a threshold optical density was obtained that discriminated staining from background. Each region of interest was manually edited to eliminate artifacts. For Aβ plaque burden analysis, data is reported as a percentage of the immunolabeled area (positive pixels) relative to the full area captured (total pixels). Each analysis was done by a single investigator blind to sample identities.

### Analysis of Aβ levels

The levels of Aβ_40_ and Aβ_42_ in the hippocampi of mice from protocol 2 were quantified by enzyme-linked immunosorbent assay (ELISA). To assess soluble Aβ levels, brain tissues were homogenized in radioimmunoprecipitation assay (RIPA) buffer, centrifuged at 20,000 × *g* for 30 min and the supernatants were stored at −80°C until use. To determine insoluble Aβ, the detergent-insoluble pellets were homogenized by sonication in a 5 M guanidine HCl (pH 8.0) solution (Sigma-Aldrich), incubated for 3 h in a rocking platform at room temperature and centrifuged at 20,000 × *g* for 30 min at 4°C. The supernatants were stored at −80°C until use or immediately diluted in PBS with 5% bovine serum albumin (Sigma-Aldrich) and 0.03% Tween-20 supplemented with 1× protease inhibitor cocktail (Roche Molecular Biochemicals, Indianapolis, IN). These brain extracts were used for Aβ analysis using ELISA kits (Invitrogen, Carlsbad, CA) according to the manufacturer's recommendations.

### Western blot

Brain tissues from mice in protocol 1 were analyzed by Western blot as previously described (Echeverria et al., 2009). Brains were rapidly removed and tissues dissected and disrupted by sonication in RIPA buffer (Cell Signaling Technology, Danvers, MA) with a complete protease inhibitor cocktail (Roche Molecular Biochemicals). Brain extracts were centrifuged at 20,000 × *g* for 30 min at 4°C. Equal amounts of protein from the supernatant were separated by electrophoresis using 4–20% Tris-Glycine gel (Thermo Fisher Scientific, Rockford, IL). The separated proteins were transferred to nitrocellulose membranes. The membranes were blocked in Tris-buffered saline with 0.05% tween 20 (TBST; Bio-Rad, Richmond, CA) containing 10% dry skim milk and incubated in TBST with primary antibodies and LI-COR's goat IRDye secondary antibodies (LI-COR Biosciences, Lincoln, NE). Rabbit polyclonal antibodies directed against PSD95 and phospho-Akt (Ser 473) were obtained from Cell Signaling Technology. A monoclonal mouse antibody against β-tubulin (Promega, Madison, WI) was used to control protein loading and transfer efficiency. Images were acquired using an Odyssey Infrared Imaging System (LI-COR Biosciences) and analyzed using the NIH Image J software.

### Statistical analysis

To analyze the group and treatment effects, any violations of the assumptions of parametric testing were first determined using the Shapiro–Wilk's test. Differences of between-groups means in the behavioral studies were assessed using One-Way and Two-Way analysis of variance (ANOVA) with *post hoc* Tukey or Tukey-Kramer (where applicable) multiple comparison tests used to identify differences between individual groups. Student's *t*-tests were used when comparing two conditions, such as the PT and Aβ plaque burden analyses. Western blot data were analyzed using the Kruskal–Wallis test and were followed by Dunn's *post-hoc* test when appropriate. Differences were considered significant with *p* < 0.05.

## Results

### Treatment with cotinine improved spatial working memory in Tg6799 mice at more advanced stage of the disease

Tg6799 mice and NT littermates began treatment at 4.5–5 months of age with vehicle (PBS, pH 7.4) or Cot 5 for which continued daily for 3 months. Then, under continued treatments, the effects of cotinine and genotype on spatial and working memory in the RAWM test were investigated using 2 (genotype) × 2 (treatment) factorial ANOVAs for both errors and latency to find the submerged platform (Figure 1A). Four experimental groups were tested: (1) vehicle-treated Tg6799 group (*n* = 8), (2) vehicle-treated NT group (*n* = 8), (3) cotinine-treated Tg6799 group (*n* = 8), and (4) the cotinine-treated NT group (*n* = 7). In the cotinine-treated NT group, one animal had sustained injury unrelated to RAWM testing or treatment, this mouse was omitted from the analysis.

The results show that Tg6799 mice were significantly impaired as compared to NT groups, making more errors across all 8 days [significant main effect of genotype: *F*_(1, 27)_ = 19.83; *p* < 0.0001; Figure 1B] and in the final 3 days of testing (final block, days 6–8) [*F*_(1, 27)_ = 15.52; *p* = 0.0005; Figure 1D], which is considered to be more relevant when assessing working memory performance. Furthermore, the latency to find the hidden platform was also significantly affected by genotype across all 8 days [*F*_(1, 27)_ = 5.343; *p* = 0.0287; Figure 1C] and in the final block of testing [*F*_(1, 27)_ = 6.990; *p* = 0.0135; Figure 1E].

Cotinine-treatment improved working memory performance in Tg6799 mice as evidenced by fewer errors to find the submerged platform across all 8 days [significant main effect of treatment: *F*_(1, 27)_ = 4.954; *p* = 0.0346; Figure 1B] and in the final block [*F*_(1, 27)_ = 6.196; *p* = 0.0193; Figure 1D]. However, there were no significant main effects of treatment detected in the analyses over latencies to find the platform across all 8 days or in the final block (Figures 1C,E).

Significant interactions between genotype and treatment levels were uncovered in the analysis of number of errors finding the platform across all 8 days [*F*_(1, 27)_ = 13.19; *p* = 0.0012]. A Tukey–Kramer's *post-hoc* test revealed that this was likely due to a cotinine-induced normalization of RAWM performance in Tg6799 mice as there were no differences between cotinine-treated Tg6799 mice and the vehicle- or cotinine-treated NT mice, with NT groups not differing from one another either. On the other hand, vehicle-treated Tg6799 mice made more errors than vehicle-treated NT mice (*p* < 0.001), cotinine-treated NT mice (*p* < 0.0001), and their cotinine-treated, Tg6799 counterparts (*p* < 0.01; Figure 1B). This was equally true when considering errors in the final block [interaction: *F*_(1, 27)_ = 6.726; *p* = 0.0152] as cotinine-treated Tg6799 mice differed only from vehicle-treated Tg6799 mice (*p* < 0.01; Figure 1D). Vehicle-treated Tg6799 mice however, did differ from both NT groups (both: *p* < 0.001) and NT groups did not differ from one another.

Taken together, these results confirm that cotinine normalized the effects of AD-like pathology on spatial and working memory performance in the RAWM task, which here, is more advanced than in our previous findings (Echeverria et al., 2011).

### Effect of cotinine on Aβ burden in the brains of Tg6799 mice

Aβ plaques begin to present in Tg6799 mice around 2 months of age and progressively increase to be full development at 8 months of age (Oakley et al., 2006). We have previously shown that cotinine prevented the development of these plaques when treatment begins prior to the onset of pathology development in Tg6799 mice (Echeverria et al., 2011). To investigate whether cotinine could reduce Aβ plaque deposition at more advances stages of the disease, Tg6799 mice at 4.5–5 months of age were evaluated for Aβ plaque burden in the hippocampus after 2 months of treatment (6.5–7 months of age at euthanization).

Tg6799 mice showed a robust Aβ plaque burden of 6.5 ± 0.8% in the hippocampus and 9 ± 0.6% in the entorhinal cortex. Compared to the vehicle-treated Tg6799 controls, cotinine-treated Tg6799 mice exhibited significant lower levels of Aβ burden in both the hippocampus (−47%; *t* = 10.03; *p* < 0.001) and entorhinal cortex (−45%; *t* = 16.47; *p* < 0.001) (Figures 2B–D).

### Effect of cotinine on Aβ levels in the brains of Tg6799 mice

We have also previously shown that treatment with cotinine (2.5 mg/kg) prior to Aβ plaque development (2 months of age) decreased Aβ plaque burden, the levels of Aβ_42_, and the ratio of Aβ_42_/Aβ_40_ in the detergent-insoluble fractions of the brains of Tg6799 mice (Echeverria et al., 2011). In this study, we investigated whether a two-fold higher dose of cotinine (5 mg/kg) could reduce Aβ_42_ levels in mice when administered at more advance stages of the pathology (4–5 months of age, *n* = 7–8 per group).

In agreement with our previous findings, we found that the levels of Aβ_42_ in the insoluble fraction were substantially higher than Aβ_40_ levels in the hippocampus of Tg6799 mice (Table 1). Insoluble Aβ_42_ levels were decreased in both the hippocampus (−29%, *t* = 1.59, *p* = 0.14) and cortex (−38%, *t* = 2.1, *p* = 0.056) of cotinine-treated Tg mice when compared to vehicle-treated Tg6799 mice. A significant decrease in the levels of insoluble Aβ_40_ was found in the hippocampus (*t* = 0.447, *p* = 0.047), and in the cortex (−56%, *t* = 2.51, *p* = 0.03).

**Table 1:** **Levels of soluble and insoluble Aβ in the hippocampus and frontal cortex of Tg6799 mice**.

	**Soluble (ng/mg)**			**Insoluble (ng/mg)**		
**Hippocampus**	**Aβ_42_**	**Aβ_40_**	**Aβ_42_/Aβ_40_**	**Aβ_42_**	**Aβ_40_**	**Aβ_42_/Aβ_40_**
Vehicle	63 ± 9	29 ± 4	2.5 ± 0.7	1315 ± 124	95 ± 12	66 ± 18
Cotinine	25 ± 11^*^	20 ± 7	1.2 ± 1.0^*^	940 ± 191	57 ± 13^*^	52 ± 13
**Cortex**	**Aβ_42_**	**Aβ_40_**		**Aβ_42_**	**Aβ_40_**	**Aβ_42_/Aβ_40_**
Vehicle	<0.015	<0.008		784 ± 53	36 ± 7	27 ± 6
Cotinine	<0.015	<0.008		492 ± 129^†^	16 ± 4^*^	21 ± 5

*The data is expressed as the mean ± SD in ng/mg of wet tissue from 6 to 8 mice/condition. Student's t-test was used to compare the mean of the values between groups*.

* *p* < 0.05;

† *p = 0.056*.

In the hippocampus of cotinine-treated Tg6799 mice, with respect to Tg controls, we found a significant decrease in the levels of soluble Aβ_42_ (−54%, *t* = 2.21, *p* = 0.049) but a non-significant difference in soluble Aβ_40_ peptide levels (+31%, *t* = 0.02, *p* = 0.98).

The levels of soluble Aβ peptides in the cortex of Tg mice were undetectable. A consistent decrease in the ratio of Aβ_42/40_ in the soluble (−52%, *t* = 2.73, *p* = 0.02) and insoluble fractions of the hippocampus (−22%, *t* = 0.68, *p* = 0.51) was found. Similarly, a decrease in the Aβ_42_/Aβ_40_ ratio was observed in the cortices of Tg mice, but this difference did not reach significance (−22%, *t* = 0.79, *p* = 0.45).

### Cotinine decreased depressive-like behavior in Tg6799 mice

It has been previously shown that in addition to apathy, AD patients have a high incidence of depression (Chung and Cummings, 2000). Higher levels of depressive-like behavior have also been observed in Tg AD mice, expressed as increased immobility in the PT (Filali et al., 2009; Hou et al., 2010). Here, we show that untreated 4.5–5 month-old Tg6799 mice displayed significantly greater immobility times than NT littermates in the PT (*t* = 2.158, *df* = 30, *p* = 0.0391; Figure 3B). We have previously shown that cotinine reduces depressive-like behavior in C57BL/6 mice following 1 week of treatment (Grizzell et al., 2014). However, nothing is known regarding cotinine's effects on depressive-like behavior in AD conditions. Following 10 days of treatment with cotinine (5 mg/kg) or vehicle in Tg6799 mice, cotinine significantly reduced the immobility times of Tg6799 mice (*t* = 2.775, *df* = 14, *p* = 0.0149; Figure 3C).

### Cotinine stimulates the pAkt/PSD95 pathway in the hippocampus and frontal cortex of Tg6799 mice

**Figure 4:**
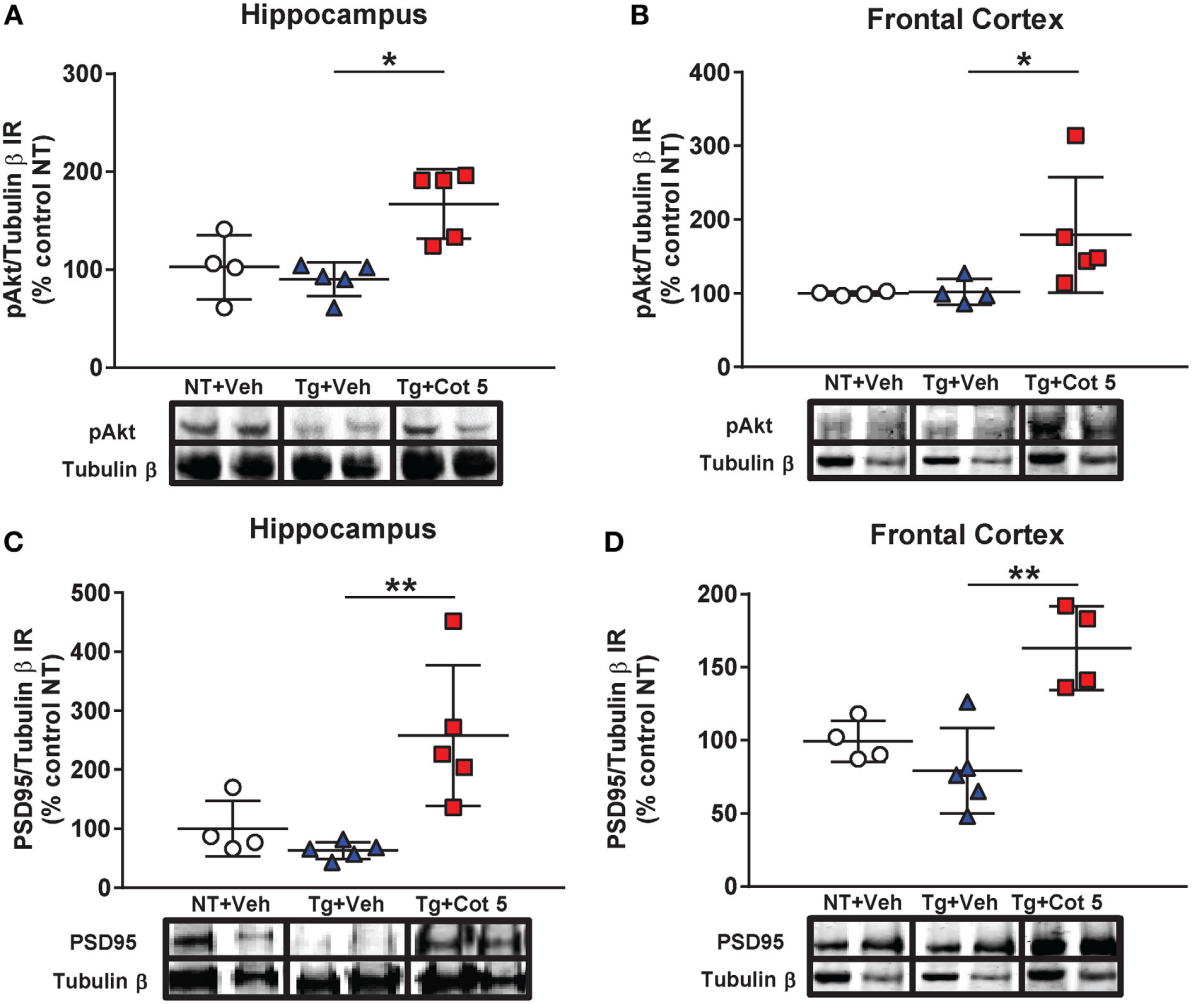
**Cotinine stimulated the Akt/PSD95 pathway in the hippocampus and frontal cortex of Tg6799 mice.** Western blot analysis of the immunoreactivity (IR) for phosphorylated Akt (pAkt), PSD95 and tubulin β in the hippocampus and frontal cortex of vehicle-treated non-transgenic (NT) control mice (NT + Veh), vehicle-treated transgenic (Tg) mice (Tg + Veh), and cotinine-treated Tg mice (Tg + Cot 5). Tg + Cot 5 mice had significantly greater levels of pAkt (^*^p < 0.05; **A**) and PSD95 (^**^p < 0.01; **C**) than Tg + Veh counterparts in the hippocampus. Similarly, Tg + Cot 5 mice showed higher levels of pAkt (^*^p < 0.05; **B**) and PSD95 (^**^p < 0.01; **D**) than Tg + Veh controls. The scatter plots with mean and standard deviation represent the IR of pAkt and PSD95 normalized against tubulin β values and expressed as percentage of NT control values. Micrographs of the Western blot images are also included.

PSD95 is a synaptic protein and its expression is required for NMDA-dependent synaptic plasticity. Furthermore, the expression of PSD95 is decreased in the brains of individuals with mild cognitive impairment (Sultana et al., 2010). PSD95 expression can be induced by activation of Akt signaling. Since we have previously found that cotinine activated Akt, we investigated whether cotinine could trigger PSD95 expression in the hippocampus and frontal cortex. The results of the Kruskal–Wallis test showed statistically significant differences in Akt phosphorylation (pAkt) between groups in the hippocampus [*H*_(2, 14)_ = 8.197, *p* = 0.0074] and frontal cortex [*H*_(2, 13)_ = 7.853, *p* = 0.0077]. A Dunn's *post-hoc* test showed a significant increase in pAkt in the hippocampus (*p* < 0.05; Figure 4A) and prefrontal cortex (*p* < 0.05; Figure 4B) of cotinine-treated Tg6799 mice when compared to vehicle-treated Tg6799 mice.

In addition to those observed in pAkt, statistically significant differences in PSD95 expression were found in the hippocampus [*H*_(2, 14)_ = 9.686, *p* = 0.0006] and frontal cortex [*H*_(2, 13)_ = 8.782, *p* = 0.0021] by the Kruskal–Wallis test. A Dunn's *post-hoc* test showed a significant increase in PSD95 in the hippocampus (*p* < 0.01; Figure 4C) and prefrontal cortex (*p* < 0.01; Figure 4D) of cotinine-treated Tg6799 mice when compared to vehicle-treated Tg6799 mice.

## Discussion

Alzheimer's disease (AD) is a progressive neurodegenerative disease leading to memory and synaptic loss as well as non-cognitive psychiatric symptoms. Because AD is characterized by a deficit of cholinergic function, several cholinergic drugs have been tested including nicotine. Nicotine improved cognitive processes such as attention in rodents (Decker et al., 1992) and may be neuroprotective against Aβ toxicity (Brown et al., 2013). The activation of the α7 nicotinic acetylcholine receptor/phosphatidylinositol 3-kinase (α7nAChR/PI3K) signaling pathway and a cross talk with the Wnt signaling pathway seems to mediate nicotine's positive effects on memory impairment in TgAPPswe/PS1dE9 mice (Inestrosa et al., 2013). However, another report showed that nicotine potentiated the depressive actions of Aβ_1−40_ on long-term potentiation in the CA1 region of the hippocampi in rats (Freir and Herron, 2003). Furthermore, in clinical trials, nicotine improved attention but did not show memory enhancing effects in AD patients (Jones et al., 1992). In addition, many concerns exist about the risks of using a potentially hazardous chemical to treat AD patients (Baldinger and Schroeder, 1995). Similarly, literature surveying the effect of nicotine on depressive-like behavior is ambivalent with some preclinical studies reporting decreased (Djuric et al., 1999; Semba and Wakuta, 2008) or increased (Hayase, 2007, 2008, 2011, 2013) depressive behavior with nicotine use.

We have previously shown that cotinine, generated in the body as a result of nicotine metabolism (Moran, 2012), prevented cognitive impairment, diminished Aβ plaques, and Aβ oligomer levels in the brains of (5 × FAD) Tg6799 mice (Echeverria et al., 2011).

In this report, we investigated the effect of cotinine on the progression of AD-like pathology and depressive-like behavior in the same strain of AD model mice but at a more advanced stage in the pathology's development. Despite this fact, cotinine attenuated working memory impairment, even statistically normalizing this performance to the levels of both NT groups. Cotinine also decreased Aβ plaque burden in both the entorhinal cortex and hippocampus as well as decreased levels of both insoluble and soluble Aβ_40_ and Aβ_42_ peptides in the hippocampus of these Tg6799 mice. These positive effects were also accompanied by the stimulation of the Akt/PSD95 pathway in the hippocampus and frontal cortex of these mice. In addition, cotinine reduced depressive-like behavior in Tg6799 mice, which extends our previous findings of the generic antidepressant effects of cotinine in normal and chronically stressed C57BL/6 mice (Grizzell et al., 2014) to subjects with AD-like symptoms.

Neuropsychiatric disturbances such as depression are critical in primary care giving of AD patients. Growing evidence suggests that depression may be both a cause and a consequence of AD and that antidepressants may be useful against this condition (Hou et al., 2010). There are several hypotheses regarding the cause of depression in AD, including emotional stress (Meynen et al., 2007), neuroinflammation, and neurodegeneration (Aznar and Knudsen, 2011). A decrease of serotonin (5-HT) in the brain is considered one of the possible causes of depression in AD (Modrego, 2010). Previous studies of non-cognitive behavioral changes in AD model mice showed that they present neuropsychiatric symptoms such as aggression (Pugh et al., 2007; Vloeberghs et al., 2007), anxiety (Vloeberghs et al., 2007), and depressive-like behavior (Filali et al., 2009) similar to the symptoms observed in humans. For example, one of these studies has shown that TgAPPswe/PS1dE9 mice have higher levels of irritability, poorer nest building, and higher immobility times in the PT compared to NT control littermates (Filali et al., 2009). Similarly, we observed an increase in immobility in the PT in Tg6799 mice prior to any intervention. Following 10 days of treatment, however, the cotinine-treated mice displayed lower levels of immobility, which was not attributable to cotinine-induced changes in locomotor activity (Zeitlin et al., 2012; Grizzell et al., 2014). The observed decrease in depressive-like behavior induced by cotinine is intriguing considering that depression is the main non-cognitive symptom observed in AD (Aznar and Knudsen, 2011; Barber, 2011), and that current antidepressants typically fail to bolster its detrimental effects in these patients (Modrego, 2010; Weintraub et al., 2010) likely due to an alternative etiology or mechanism underlying its development from non-AD sufferers (Hollingworth et al., 2006). It has been demonstrated that cotinine increases 5-HT levels in the rat brains (Fuxe et al., 1979) and it is therefore possible that the antidepressant effects of cotinine are partially mediated by an increase in 5-HT levels in the brain. Also, 5-HT does exert positive effects on memory, seemingly mediated by a subset of 5-HT receptors such as 5-HT_1A_, 5-HT_4_, and 5-HT_6_ (King et al., 2008). Although these are plausible ideas, selective serotonin reuptake inhibitors (SSRIs) have not shown beneficial effects in AD patients in clinical trials (Modrego, 2010). Thus, some of the beneficial effects of cotinine could be due to the stimulation of serotonergic activity but may act through additional mechanisms which facilitate and supplement its antidepressant and pro-cognitive effects in AD model mice.

AD is characterized by deficits of memory processes (Stone et al., 2011). This has been mimicked in animal models of the disease as well (Oakley et al., 2006; Ohno et al., 2006). We have previously shown that when administered prior to Aβ plaque pathology development in Tg6799 mice, at 2 months of age, cotinine-treatment prevented AD-induced impairment of working memory in the RAWM task, a powerful measure of working memory in rodents (Echeverria et al., 2011). When treatment began at more advanced stages of this disease (~5 months), the current results show that cotinine treatment also normalized the performance in the RAWM to the levels of NT animals at an age when Tg6799 mice would present abundant Aβ plaque pathology (~8 months) and cognitive impairment (Oakley et al., 2006). This is particularly encouraging as memory impairments are at the forefront of AD symptomology. Furthermore, despite recent advances in detecting early Aβ plaque pathology, it is not always possible to identify individuals who will suffer from dementia and AD prior to the disease's development, therefore finding effective therapies in reducing cognitive impairment after the pathology has advanced to middle stages is crucial.

Episodic memory involves the capacity to learn, store, and retrieve information about experiences such as when (time), where (place), and what (event). AD patients in early stages of the pathology present with severe deficits in episodic memory. The circuits supporting episodic memory are highly conserved across mammalian species from rodents to primates and involves neocortical association areas, the parahippocampal region, and the hippocampus (Eichenbaum, 2006). RAWM test is broadly used to determine memory deficits in transgenic AD mice. RAWM is dependent on hippocampus activity, and it is generally accepted to be a sensitive test for short-term episodic memory (Savonenko et al., 2005).We have previously shown that cotinine administered at early stages of the pathology prevented short-term episodic and reference memory impairment in Tg6799 mice. Here, we report that depressive-like behavior in Tg6799 mice appears early during the pathology and continues at more advanced stages. In our model, cotinine reduced depressive-like behavior at all stages of the pathology. Thus, it is reasonable to speculate that the prevention of memory loss by cotinine in our first study may also involve a decrease in depressive-like behavior at early stages of the pathology. However, further experiments are required to confirm this idea.

In addition to its enhancement of cognitive and non-cognitive symptoms, cotinine decreased Aβ plaque burden and the levels of Aβ_40/42_ peptides in the brains of Tg6799 mice. These effects mirrored the observed decrease in Aβ plaques and levels of soluble Aβ peptides accumulating in the brain induced by pretreatment with cotinine (2.5 mg/kg) (Echeverria et al., 2011). This time, when mice were treated at later stages of the disease, the beneficial effect in reducing plaque pathology was higher than in the previous study. In our previous study, a 5-month cotinine (2.5 mg/kg) treatment beginning at 2 months of age resulted in reduced Aβ plaque size at an average of 26 and 17% in the cingulate and motor cortices, respectively (Echeverria et al., 2011). Instead, when treatment was delayed until ~5 months of age, 2 months of treatment with cotinine (5 mg/kg) reduced Aβ plaque burden by 47 and 45% in the hippocampus and entorhinal cortex, respectively. This enhanced effect may be the result of the increased dosage used. However, it is important to consider that at later stages of AD-like pathology, the rate of Aβ plaque burden progression is much faster. Thus, we would expect to observe higher differences in percentage of Aβ plaque burden with a drug that halts the aggregation of the Aβ peptides at middle stages of the disease. Also, we may consider that the size of the Aβ plaques at the age tested in both studies (~8 months of age) were lower in the cingulate and motor cortices than the observed in the hippocampus and entorhinal cortex in Tg6799 mice. This difference further complicates the interpretation as a lower development of Aβ plaques in the former regions will result in a slower building of Aβ plaques, thus resulting in lower differences with the mice treated with cotinine (2.5 mg/kg) beginning at 2 months. Independent of the previous considerations, the results clearly showed that cotinine can not only slow down or prevent Aβ plaque development when it is administered at early stages of the disease, but can also halt its progression at later stages. Further studies are required to assess whether cotinine may also facilitate the clearance of the Aβ plaques. On the other hand, the analyses of the Aβ levels show that treatment with cotinine at the later stage, even at a higher dose, results in higher levels of insoluble and soluble Aβ_42_ peptides than mice treated at the early stage of the disease (Echeverria et al., 2011). It is interesting that in spite of these higher levels of Aβ peptides in the hippocampus, a significant positive effect over cognition was still attained in the cotinine-treated mice when compared to vehicle-treated mice. This permits us to suggest that cotinine may have an additional mechanism supporting its neuroprotective effect other than reducing Aβ aggregation.

Neuroinflammation and a decrease of neurotrophic factors levels in the brain are considered key factors inducing synaptic loss and a common link between depression and AD (Wuwongse et al., 2010). Morphological studies have shown that a significant correlation exists between memory performance and synaptic plasticity during memory consolidation, and the loss of synaptic spines correlates with cognitive impairment in AD (Pozueta et al., 2013). Therefore, the enhancement of synaptic function may be fundamental to restore cognitive abilities and mood in neurodegenerative conditions, including AD (Yu and Lu, 2012). We have previously shown that cotinine induces increases in synaptophysin, a synaptic protein serving as a marker for synaptic density, and decreased depressive-like behavior induced by chronic stress in mice (Grizzell et al., 2014). Consistent with this idea, we found that cotinine diminished depressive-like behavior, increased cognitive abilities, and enhanced the expression of synaptic protein PSD95 in the hippocampus and frontal cortex of Tg6799 mice. PSD95 plays a key role promoting brain plasticity by controlling NMDA receptor signaling (Wang et al., 2012). A previous study showed that extracellular signal-regulated kinase (ERK)-dependent PSD95 induction in the gustatory cortex was an essential step in taste learning (Elkobi et al., 2008). A more recent study has shown that other natural products such as the ginkgo flavonols, quercetin, and kaempferol, stimulated the brain-derived neurotrophic factor (BDNF)/cyclic AMP response element binding protein/PSD95 pathway and reduced Aβ in neurons isolated from TgAPPswe/PS1dE9 mice (Hou et al., 2010). The synthesis of PSD95 is a process that occurs at the synapse and its mRNA is abundant in the neuropil regions along with other dendritic proteins such as dendrin and the microtubule-associated protein 1A (Cajigas et al., 2012). It has been shown that BDNF promotes translation of specific mRNAs including PSD95 by regulating the activity of the protein synthesis machinery.

Expression of long-lasting synaptic plasticity and long-term memory requires protein synthesis, especially since some mRNAs of synaptic proteins are already located at the synapse waiting for translation at the ribosome. The translation of PSD95 is promoted by the phosphorylation of the modulators of protein translation eukaryotic translation initiation factor 4E (eIF4E) and eukaryotic translation initiation factor 4E binding protein 1 (4EBP1) by activating the ERK and PI3K/Akt signaling pathways, respectively (Leal et al., 2014). PI3K/Akt/mTOR (mammalian target of rapamycin)-cAMP response element-binding protein (CREB) and ERK-dependent signaling pathways modulate the protein translation efficiency necessary for establishing long-term synaptic plasticity (Kelleher et al., 2004). Alterations in Akt phosphorylation, mTOR activity and its downstream targets 4E-BP1, eukaryotic elongation factor 2 (eEF2), and eEF2 kinases have been found in AD brains (Li et al., 2005). Although it is controversial, it has been reported that there is a decrease in mTOR signaling in the brains of AD mice (Caccamo et al., 2013; Gouras, 2013; Tang et al., 2013) and AD patients (Lafay-Chebassier et al., 2005; Pei and Hugon, 2008; Sun et al., 2014). Moreover, the inhibition of mTOR signaling correlates with impairment in synaptic plasticity in hippocampal slices from an AD mouse model and in wild-type slices exposed to exogenous Aβ. The activation of mTOR may rescue cognitive impairment, as its up-regulation through GSK3 inhibitors rescued long-term potentiation, a cellular model of synaptic plasticity, in a mouse model of AD (Ma et al., 2010). The authors concluded that the mTOR pathway modulates Aβ-related synaptic dysfunction in AD (Ma et al., 2010).

Our data confirms previous results showing that cotinine activates Akt in the hippocampus and showed for the first time that this activation corresponds with an increase in the expression of PSD95 in the brain of Tg6799 mice. Our results are also in agreement with another study showing that antidepressants increased PSD95 in the hippocampi of TgAPPswe/PS1dE9 mice (Hou et al., 2010).

Cotinine-induced Akt activation can be the outcome of the enhancement of cholinergic neurotransmission, which would favor synaptic function. The α7 and α4β2 nAChRs are the most abundant nicotinic receptors in the brain. These nicotinic receptors are broadly expressed in brain regions such as the amygdala, prefrontal cortex, hippocampus, hypothalamus, and striatum involved in emotional responses, learning, and memory. Furthermore, nAChRs may influence depressive behavior by controlling dopaminergic, serotoninergic, glutamatergic, and γ-aminobutyric acid neurotransmission. A deficit in nicotinic receptors is considered a key player in AD pathophysiology (James and Nordberg, 1995). AD patients have a reduction in cortical nicotinic cholinergic receptor when compared to age-matched control individuals (Flynn and Mash, 1986; Whitehouse et al., 1986; Aubert et al., 1992; Freedman et al., 1995).

Cotinine is a poor agonist of nAChRs but may act as a positive allosteric modulator (PAM) of α7nAChRs. As a PAM, cotinine will not have agonistic effect over the receptors but will enhance its activation by agonists (Oddo, 2012). This idea explains recent evidence that both the improvement of sensorimotor desensitization (Vainio et al., 2000; Wildeboer-Andrud et al., 2014) and fear extinction induced by cotinine (de Aguiar et al., 2013) depended on the activation of α7 and α4β2nAChRs subtypes. In addition, previous evidence suggests that cotinine, by stimulating the α4β2nAChRs and/or α6β2nAChRs, may evoke the release of DA in a calcium-dependent manner in the striatum (Dwoskin et al., 1999). Since the striatum is part of the neuronal network supporting working memory (Spencer et al., 2012), the stimulation of dopamine release can be an additional mechanism by which cotinine may be influencing learning and memory.

A recent study highlighted the importance of the stimulation of calcium signaling through α7nAChRs (Cheng and Yakel, 2014). The presence of α7nAChRs and the effect of its stimulation in mossy fibers on the hippocampus stimulation were investigated. The authors expressed the genetically encoded calcium indicator GCaMP3 in dentate gyrus granule cells and used a PAM of α7nAChRs to diminish the desensitization of the receptor and enhance calcium signaling. By comparing calcium responses in wild-type and α7nAChR knock-out mice, they demonstrated that α7nAChR-dependent calcium currents enhanced glutamate release at the mossy fiber terminals projecting to the CA3 region of the hippocampus (Cheng and Yakel, 2014). The importance of the stimulation of increased intracellular calcium levels by α7nAChRs for neurotransmitter release probability was also indicated by paired-pulse facilitation plasticity studies.

**Figure 5:**
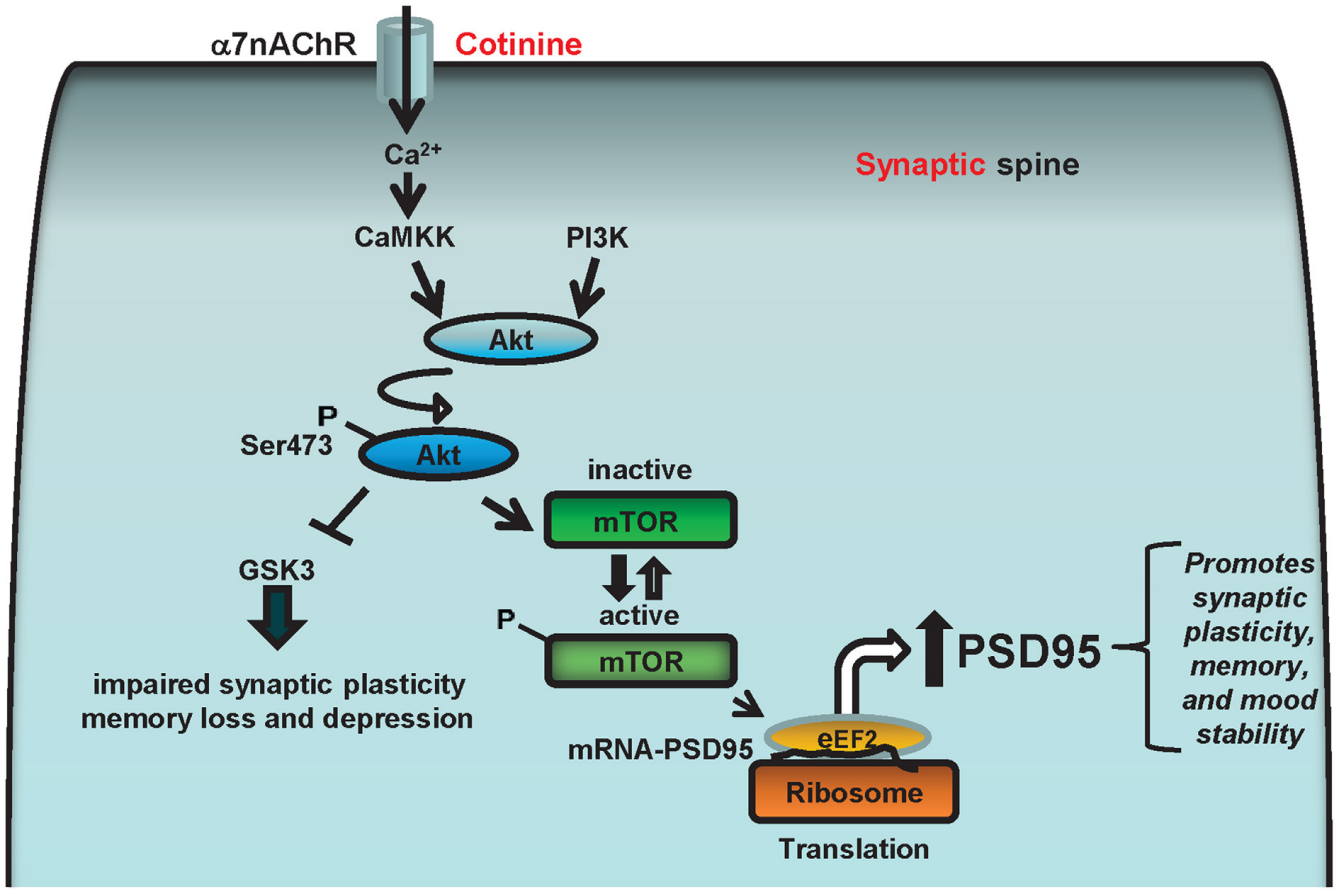
**Diagram representing the potential cell signaling pathways underlying the positive actions of cotinine on synaptic plasticity and behavior.** The α7nAChRs can stimulate Akt (aka, protein kinase B) through the calcium-dependent activation of CaMKK, which can activate Akt that in turn phosphorylates and stimulates CREB and/or the mammalian target of rapamycin (mTOR) activity. The α7nAChR may also stimulate Akt through ERK/PI3K, and CREB and mTOR phosphorylation. mTOR, and CREB are serine threonine kinase, that regulate synaptic plasticity and memory formation. Also, mTOR positively controls the translational machinery to synthesize synaptic proteins such as PSD95 by stimulating the phosphorylation of the protein translation modulators eIF4E and 4EBP1. Therefore, cotinine may enhance the translation of PSD95 by activating ERK/PI3K/Akt and/or CamKK/Akt signaling pathways *via* positively modulating α7nAChR.

Based on this evidence, we hypothesize that cotinine facilitates α7nAChR-mediated increase in calcium concentration which stimulates Akt activation by PI3K and/or calcium/calmodulin-dependent protein kinase kinase (CaMKK). Once activated, Akt can inhibit GSK3β and phosphorylate mTOR or CREB, favoring the expression of PSD95 at the synapse as well as reducing synaptic loss. The increase in PSD95 will have a positive effect on synaptic plasticity underlying learning and memory processes as well as mood stability. Thus, a cotinine-induced increase in the translation of synaptic proteins at the synapse may prevent or halt the deficits in synaptic plasticity in AD brains, simultaneously improving both cognitive abilities and mood in Tg6799 mice (Figure 5).

Overall, we showed that cotinine halted the cognitive and depressive-like symptoms of AD at later stages of the disease's progression. The beneficial actions of cotinine were accompanied by the stimulation of the Akt/PSD95-CREB pathway in AD brains. Since this pathway promotes synaptic plasticity and prevents synaptic loss, we conclude that cotinine may prevent and reduce AD pathology by mitigating synaptic dysfunction in the hippocampus and frontal cortex in Tg6799 mice. These results are related to a model of FAD linked to genetic mutations in both the APP and the PS1 genes. The Tg6799 mouse model of AD develops Aβ plaques and several behavioral aspects of the disease, including working memory deficits and depressive-like behavior as well as presents neurochemical changes observed in AD brains. However, more than 95% of AD cases are sporadic (SAD) with a late onset and <5% of AD cases are FAD with an early onset. The causes of SAD are not well understood, thus, it is possible that the observed neurochemical changes between SAD and FAD may differ. However, the cholinergic and synaptic deficits are common characteristic to both SAD and FAD and the changes induced by cotinine through the improvement of cholinergic function and nAChRs signaling are likely to occur in both SAD and FAD. More importantly, brain senescence is a common link between both types of AD. The senescence hypothesis links the progression of AD to changes in energy metabolism, Ca^2+^ deregulation, apoptosis (Mattson, 2007), and neuroinflammation (Verri et al., 2012).

Cotinine can be an excellent therapeutic agent alone or in combination with other current or future medications because it has minor side effects in humans. Considering the increasing necessity and world-wide demand of new drugs to prevent, slow down, or ideally, halt AD, the testing of cotinine in well-designed clinical studies is urgently needed.

## Author contributions

Sagar Patel and Valentina Echeverria contributed to the design and conceptualization of the study, the analysis and interpretation of the data, and drafting and revising the manuscript for intellectual content. J. Alex Grizzell, Rosalee Holmes, Ross Zeitlin, Rosalynn Solomon, Thomas L. Sutton, Adeeb Rohani, Laura C. Charry, Alexandre Iarkov, and Takashi Mori contributed to the analysis and interpretation of the data, and revising the manuscript for intellectual content.

### Conflict of interest statement

Sagar Patel, J. Alex Grizzell, Rosalee Holmes, Ross Zeitlin, Rosalynn Solomon, Thomas L. Sutton, Adeeb Rohani, Laura C. Charry, Alexandre Iarkov, and Takashi Mori have no actual or potential conflict of interests concerning the research in the present paper. Valentina Echeverria Moran is the inventor of a pending patent application from the University of South Florida and the Veterans Affairs administration (US 20100104504).

## Abbreviations

Aβ: amyloid-β
AChE: acetylcholinesterase
AD: Alzheimer's disease
Akt (PKB): protein kinase B
APP: amyloid precursor protein
ANOVA: analysis of variance
BDNF: brain-derived neurotrophic factor
CaMKK: calmodulin-dependent protein kinase kinase
4EBP1: eukaryotic translation initiation factor 4E-binding protein 1
ELISA: enzyme-linked immunosorbent assay
ERK: extracellular signal-regulated kinase
eIF4E: eukaryotic translation initiation factor 4E
GSK3β: glycogen synthase kinase 3β
FAD: familial AD
FS: forced swim
5-HT: serotonin
IHC: immunohistochemical
MAOIs: monoamine oxidase inhibitors
mTOR: mammalian target of rapamycin
NFT: neurofibrillary tangle
nAChRs: nicotinic acetylcholine receptors
NMDA: N-methyl-D-aspartate
NT: non-transgenic
OF: open field
PAM: positive allosteric modulator
PI3K: phosphatidylinositol 3-kinase
PS1: presenilin 1
PSD95: postsynaptic density protein 95
PT: Porsolt's forced swim test
RAWM: radial arm water maze
RIPA: radioimmunoprecipitation assay
SAD: sporadic AD
SSRIs: selective serotonin reuptake inhibitors
SNRIs: serotonin norepinephrine reuptake inhibitors
T: trial
TBST: Tris-buffered saline with 0.05% tween 20
TST: tail suspension test.
